# Micromechanical Resonator Driven by Radiation Pressure Force

**DOI:** 10.1038/s41598-017-16063-4

**Published:** 2017-11-22

**Authors:** Joseph A. Boales, Farrukh Mateen, Pritiraj Mohanty

**Affiliations:** 1 Department of Physics, Boston University, 590 Commonwealth Avenue, Boston, MA 02215 USA; 2 Department of Mechanical and Aerospace Engineering, Boston University, 110 Cummington Street, Boston, MA 02215 USA

## Abstract

Radiation pressure exerted by light on any surface is the pressure generated by the momentum of impinging photons. The associated force – fundamentally, a quantum mechanical aspect of light – is usually too small to be useful, except in large-scale problems in astronomy and astrodynamics. In atomic and molecular optics, radiation pressure can be used to trap or cool atoms and ions. Use of radiation pressure on larger objects such as micromechanical resonators has been so far limited to its coupling to an acoustic mode, sideband cooling, or levitation of microscopic objects. In this Letter, we demonstrate direct actuation of a radio-frequency micromechanical plate-type resonator by the radiation pressure force generated by a standard laser diode at room temperature. Using two independent methods, the magnitude of the resonator’s response to forcing by radiation pressure is found to be proportional to the intensity of the incident light.

## Introduction

The concept of radiation pressure was first discovered by Maxwell in the nineteenth century^1^. Even earlier in 1619, Kepler had used the notion of classical radiation pressure to explain why comet tails face away from the sun^2^. On the macroscale, it has been linked to significant perturbations in satellite orbits around earth^3–5^ and has been used to provide an additional thrust and stabilizing force for probes in transit to other celestial bodies^6–8^. If the intensity of light is sufficiently large, as with a laser, then the magnitude of the force can be comparable to or larger than other forces in the problem^9–14^, and has even been shown to be a noise source that can be comparable to thermal noise in certain systems^15^. Most recently, these small forces from radiation pressure have been used for wireless optical data transfer with nonlinear micromechanical resonators via sideband modulation^16^. Even in more highly-damped systems, MEMS resonators have been shown to be capable of easily detecting small acoustic pressures^17^. Radiation pressure has, indeed, been used in previous experiments for switching between bifurcated modes^18^, to induce stochastic resonances^19 in laser-cooling applications 20–23^, and its effects on high-finesse cavities has even been studied^24–28^. However, use of radiation pressure as the only source of a mechanical force for direct actuation of relatively large-scale objects, such as a sub-millimeter-sized mechanical resonator is yet to be demonstrated. For the first time, we demonstrate the direct actuation of a micromechanical resonator using only the force provided by radiation pressure.

Assuming that all radiation incident on a surface is either reflected or absorbed, valid for any sufficiently thick, opaque object, the force exerted by the radiation pressure of light can be written

1 $${F}_{rad}=\frac{(R+1){I}_{rad}A}{c},$$

where *R* is the surface reflectivity of the object experiencing the force, *I*_*rad*_ is the intensity of the beam, *A* is the effective area of the top surface of the resonator, and *c* is the speed of light. A time-varying force is produced by adding an RF signal on top of a constant DC signal to a standard laser diode (LD) with the beam normally incident on a micromechanical resonator such that $F(t)={F}_{DC}+{F}_{0}\,\sin \,\omega t.$

In the linear regime, the resonator is described as a damped driven harmonic oscillator

2 $$\ddot{x}+\frac{{\omega }_{0}}{Q}\dot{x}+{\omega }_{0}^{2}x=\frac{F(t)}{m}.$$

where *x* is the effective position of the resonator, *m* is the mode-dependent effective mass, *ω*_0_ is the resonant frequency, *Q* is the quality factor, and *F(t)* is the time-varying force used to drive the resonator. The frequency response of such a system can be obtained by Fourier transform:

$$|X(\omega )|=(\frac{{F}_{0}}{m})/\sqrt{{({\omega }_{0}^{2}-{\omega }^{2})}^{2}+\frac{{\omega }_{0}^{2}}{{Q}^{2}}{\omega }^{2}}.$$

For small displacements, or in the linear response regime, the signal size can be written as

$$|V(\omega )|={A}_{signal}/\sqrt{{({\omega }_{0}^{2}-{\omega }^{2})}^{2}+\frac{{\omega }_{0}^{2}}{{Q}^{2}}\,{\omega }^{2}}$$

where *A*_*signal*_ represents the mode- and force-dependent size of the driving signal.

**Figure 1:**
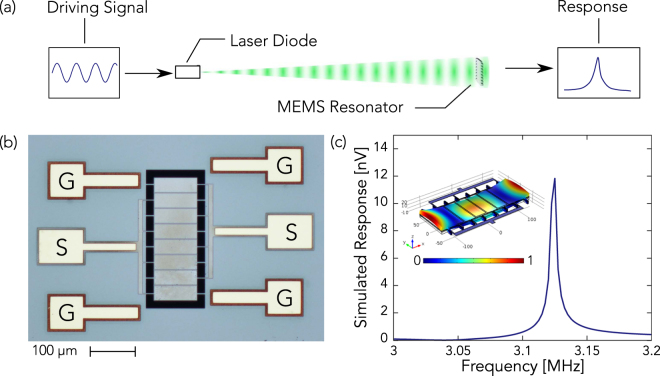
(**a**) Schematic diagram of experiment to wirelessly actuate a MEMS resonator using radiation pressure. A DC current is provided to the laser by its driver, and a RF signal is provided by a signal generator which is directly coupled to the LD through a bias-T network to modulate its intensity. The beam from the LD is normally incident on a MEMS resonator, and the signal produced by the resonator is amplified, then measured by a spectrum analyzer. (**b**) A picture of the MEMS resonator used for this experiment. The resonator is a 96-by-270-µm plate resonator suspended by sixteen legs. The electrodes marked “G” are for connection to the grounding plane, and the electrodes marked “S” are for driving the resonator or measuring its response. (**c**) The plot shows the simulated response of the resonator using COMSOL, where an isotropic loss of 0.001 is introduced to simulate damping forces. The inset illustrates the mode that is most strongly excited by a uniform radiation pressure.

In this experiment, the light beam is produced by a standard 15-mW, 520-nm LD which is modulated at RF and is normally incident on the resonator’s surface, as shown in Fig. 1(a). To produce RF modulation of the intensity, the LD is directly coupled to a RF signal generator via a built-in bias-T network; in addition, a DC current is provided to the LD by its driver. The direct coupling has a 50-ohm impedance, and the amplitude of the laser’s intensity modulation is directly proportional to the RF signal size. The resonator, shown in Fig. 1(b), is a 96-by-270-µm plate-type resonator, suspended by sixteen 15-by-3-µm anchors. From bottom layer to top layer, the device is constructed from 10-µm silicon plus 1-µm silicon dioxide structural layers, a 300-nm thick molybdenum ground electrode, a 700-nm aluminum nitride piezoelectric layer, and 300-nm-thick interdigitated molybdenum signal electrodes. Any mechanical strain in the piezoelectric layer produces an electric field which causes a potential difference between the ground and signal electrodes; this signal is amplified, then measured using a spectrum analyzer. Eigenfrequency and frequency domain simulations were performed using COMSOL to identify modes that are likely to be excited by radiation pressure and to estimate the resulting signal size. The numerical results for the mode which is the most strongly excited are shown in Fig. 1(c). These results suggest that, with amplification, actuation by radiation pressure should be experimentally measurable.

**Figure 2:**
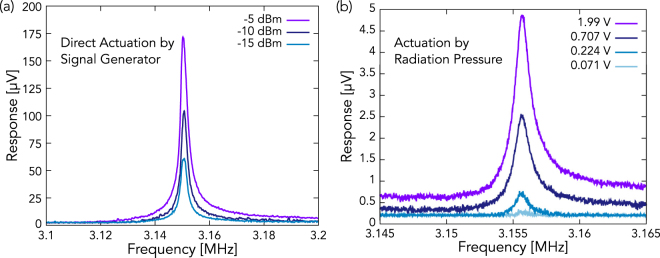
(**a**) The resonator was driven with a signal generator at one of its signal electrodes, and its response was measured at the second signal electrode using a spectrum analyzer. The different line colors represent driving powers, and, as driving power increases, so does the response size. (**b**) The resonator was driven by the radiation force of a RF-modulated laser, and its response was amplified, then measured using a spectrum analyzer. The voltages in the key represent the RMS signal size that was used to modulate the 50-Ω-impedance LD. As the modulation size increases, so does the size of the resonator’s response.

Guided by the simulation results, we characterized the resonator by driving one of its signal electrodes over a range of frequencies using a signal generator and measuring the frequency response using a spectrum analyzer. A Lorentzian response with a central frequency close to that obtained by simulation was identified. The responses for three different driving powers are shown in Fig. 2(a). The resonator was also driven at higher powers, and the expected nonlinear behavior was observed, further validating that this is a real mechanical mode. For this mode, we found that *Q* is 1980 and ${f}_{0}=\frac{{\omega }_{0}}{2\pi }$ is 3.15 MHz, close to the mode frequency obtained in the COMSOL simulation.

This mode is an ideal candidate for the measurement of small forces for several reasons. As previously mentioned, the measured signal is proportional to the effective modal area and modal mass. In general, both of these values are dependent on the number of nodes and antinodes of the representative mode. As shown in the inset of Fig. 1(c), the mode at 3.15 MHz is the lowest order drum-like mode of this resonator, and therefore has the largest possible effective area for this mode type, maximizing the force experienced as a result of the radiation pressure. In addition, this is a mode with even symmetry, which allows all parts of the interdigitated transducers to experience positive and negative potentials at the same time, increasing the signal measured at the spectrum analyzer. Finally, as will be discussed later, the effective force applied as a result of thermal Johnson noise increases with frequency and decreases with *Q*. Since this is mode has one of the lowest frequencies available with this resonator, and its *Q* value is relatively large, the signal-to-noise ratio for this mode’s response is also large.

Next, we attempted to excite the mode by using only the RF-modulated LD signal. The signal output by the resonator was amplified by a pre-amplifier with a gain of 36 dB before it was sent to the spectrum analyzer. The response is shown in Fig. 2(b) for a range of modulation amplitudes. As the modulation amplitude increases, so does the size of the resonator’s response. With amplification, a peak signal size of almost 5 µV was measured.

**Figure 3:**
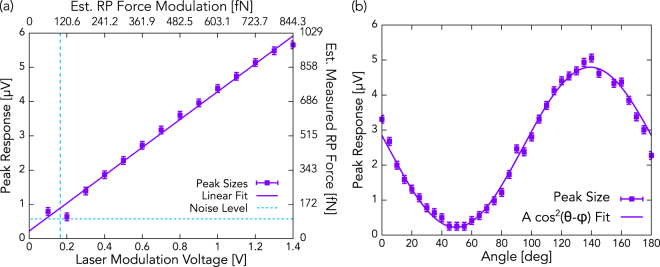
(**a**) The modulation amplitude of the LD’s intensity, which is proportional to modulation voltage provided by the signal generator, was varied to verify that the response size grows linearly with light intensity. The estimated rms force variation applied by radiation pressure is shown on the upper x-axis. The dashed line indicates the calculated level of Johnson thermal noise. (**b**) To rule out the possibility of noise being responsible for the resonant response that was observed, the intensity dependence was also measured using a polarizer. In this case, no electrical changes were made during the experiment, so any changes in response are purely a result of changes in polarization angle. The 0-degree axis was chosen arbitrarily.

To verify that it is indeed the light that produces the excitation and not some noise-related or other phenomenon, we measured the size of the peak as a function of lasing intensity using two independent methods. First, we measured the resonance amplitude for a series of different modulation amplitudes, as shown in Fig. 3(a). This method produced a linear increase in the response size with modulation voltage, as expected, due to the linear dependence of the force on intensity, described in Equation (1).

To further demonstrate that variation in intensity and hence radiation pressure force governs the driving of the resonator, we inserted a polarizer between the laser and the resonator. Light from the LD (which is already polarized) is sent through a polarizer before it reaches the resonator. As the angle of the polarizer is varied, the light intensity incident on the resonator varies as $I={I}_{rad}\,{\cos }^{2}(\theta -\varphi )$, where *I*_*rad*_ is the beam intensity produced by the LD, θ is the angle of the polarizer’s transmission axis relative to some arbitrary axis, and φ is the polarization angle of the light produced by the LD. As shown in Fig. 3(b), the peak response of the resonator does in fact vary as ${\cos }^{2}(\theta -\varphi )$. Tuning the intensity in this way prevents any changes in electrical signals and, therefore, prevents any changes in electrical noise resulting from the experimental setup. Since the resonator’s response size varies in the same manner as the intensity, we conclude that the intensity of the modulated light that is incident on the resonator is directly responsible for the resonator’s response.

While a majority of our measurements were performed using the 3.15 MHz resonance mode, we did several experiments using other resonance modes as well. These experiments were intended to show that multiple modes can be used for such measurements. In principle, *any* resonance mode should be possible to use, but those at lower frequencies with higher *Q*’s and even symmetry will more easily overcome the effects of thermal noise.

**Figure 4:**
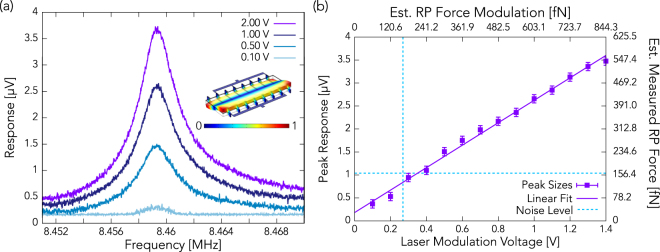
**(a)** Actuation of the 8.46 MHz mode of the resonator using radiation pressure for four different laser modulation intensities. The inset shows the modeshape, where red represents the largest relative deflection of the resonator, and blue represents the minimum deflection. **(b)** The resonance amplitude resulting from radiation pressure actuation shows a linear dependence on the laser intensity modulation amplitude. The lower x-axis shows the laser modulation potential, and the upper x-axis shows the estimated force imparted by radiation pressure. The dashed line indicates the level of Johnson thermal noise.

In Fig. 4, we show the results of the same experiment, but performed using the resonance near 8.46 MHz. This resonance has a *Q* of 2,020. The resonator’s frequency response for several different laser modulation amplitudes are shown in Fig. 4(a). These spectral response curves are once again Lorentzian in shape, as may be expected. The resonator’s peak response amplitude as a function of modulation intensity is shown in Fig. 4(b). As with the 3.15 MHz mode, the resonator’s response at 8.46 MHz is linearly dependent on the intensity.

In Figs 3(a) and 4(b), the radiation pressure force estimate was calculated by using the operational parameters of the experiment as well as the specifications provided by the equipment manufacturers. The LD is a 15-mW, 520-nm laser diode, and is transmitted into free space using a collimator, which produces a beam with width 1.48 mm (using the 1/e^2^ method). From these parameters, we calculate that the intensity at the center of the beam, *I*_*max*_, is 17.4 kW/m^2^. For these calculations, we estimate that the beam intensity is approximately constant over the surface of the resonator since the resonator is much smaller than the beam width and that the effect of the beam’s divergence is negligible since the collimator is within less than 1 meter of the resonator. The surface of the resonator is almost entirely covered with molybdenum, which has a reflectance of 0.6. Using ${F}_{rad}=(R+1){I}_{max}A/c$, we find that, at peak intensity, the laser is able to provide 1.66 pN of force. If this force is modulated at some frequency, then, as discussed earlier, the amplitude of the time-varying part of the force can be half of this value, or 831 fN for a 2-volt laser modulation voltage. The amplitude of this force is proportional to the LD modulation voltage, and an RMS modulation of 1 volt produces an amplitude of 415 fN.

In addition, we calculated the force experienced by the transducer due to radiation pressure to first-order. For this estimate, we assume the silicon and metal layers to be rigid bodies so that the only strains induced by the radiation pressure are within the AlN layer. Further, we neglect strains that are not along the c-axis of the material. For thin film AlN, the *d*_33_ coefficient, which quantifies the electric potential produced for a strain along the c-axis, is 5 pm/V^29^, and the elastic modulus *E* is 273 GPa^30^. It is trivial to show that, for a transducer with a normally applied stress (radiation pressure, in this case), the force exerted on the surface of a simple transducer is

$$F=\frac{{A}_{eff}{d}_{33}E{\rm{\Delta }}V}{t},$$

where *t* is the film thickness, and Δ*V* is the potential difference between the top and bottom of the layer. The value of Δ*V* that is shown on Figs 3(a) and 4(b) have been amplified and are enhanced by a factor of *Q*, so they are 126,720 and 129,280 times larger than the unamplified steady-state voltage, respectively. For example, a 5-µV peak in Fig. 3(a) corresponds to a 39.5-pV unamplified steady-state voltage, and to an approximate force of 1.996 pN. Adjusting for mode shape by multiplying by the ratio of average deflection and maximum deflection of the mode shape (about 43% for the 3.15 MHz mode), this reduces to 858 pN. This first-order estimate is within a factor of 2 of the results shown in Fig. 3(a), supporting our claim that we are detecting the force exerted by radiation pressure.

The thermal Johnson noise experienced by a mechanical resonator is the largest noise source in this experiment, and can be estimated as^31^

$$\langle {F}_{n}\rangle =\sqrt{\frac{4{k}_{B}TM{\omega }_{0}}{Q}},$$

where *k*_*B*_ is the Boltzmann constant, *T* is the temperature, and *M* is the effective mass of the transducer. From this expression, using the environmental conditions during the experiment along with the resonator’s properties, we can find that the thermal Johnson noise experienced by the transducer is 69.6 fN/$\sqrt{{\rm{Hz}}}$ when using the 3.15 MHz resonance. Measurements were done using a 2 Hz measurement bandwidth, so the approximate noise force during measurements was 98.5 fN. For the 8.46 MHz mode, the average force due to thermal Johnson noise is about 114.1 fN/$\sqrt{{\rm{Hz}}}$, corresponding to a noise force during measurement of 161.3 fN. These noise levels are consistent with results obtained during the experiment, as indicated by the dashed lines on Figs 3(a) and 4(b), though it is slightly overestimated for 8.46 MHz mode.

To further rule out other possible actuation sources, we performed a series of control experiments. As the DC component of the laser’s intensity varies, the resonator’s temperature similarly varies which is evident from the shifting of its resonance peak; however, changes in the amplitude of the RF component of the laser modulation have no effect on the device’s temperature. Temperature changes resulting from changes in the DC component occur on the timescale of several seconds while intensity fluctuations occur on the time scale of hundreds of nanoseconds. Finally, the resonator was strongly driven at one of its signal electrodes so that it exhibited nonlinear, hysteretic behavior. Meanwhile it was periodically heated from below using a Peltier module at a much lower frequency. The purpose of this control experiment was to produce sidebands, which are equally-spaced peaks in frequency space that appear as a result of mode mixing when a weak driving force and a much stronger driving force at different frequencies are simultaneously applied to a nonlinear resonator^16^. The weak driving force in this control experiment is in the form of a periodic thermal gradient, which should cause minor stresses in the resonator due to the induced temperature gradients. This is similar to the behavior that would be expected if the resonator was being periodically *heated* by the laser rather than periodically *pushed*. Since no sidebands occurred in the control experiment, we concluded that the effect of periodic heating by the laser is not strong enough to actuate the resonator.

The century-old quest for detection and use of radiation pressure as a standalone mechanical force^32,33^ paved the way for its ultimate use in laser cooling^34^ of much smaller systems such as atoms and ions. Pure mechanical excitation of a macroscopic, 8.7 × 10^−10^-kg resonator by radiation pressure alone, as demonstrated here, is exciting, as it may enable a novel approach to the study of quantum entanglement and quantum coherence of a macroscopic optomechanical oscillator.

In conclusion, we have demonstrated direct actuation of a micromechanical resonator using radiation pressure. By performing two independent intensity dependence measurements, we find that the resonator’s response amplitude is directly proportional to the size of the laser’s intensity modulation. In addition to being a novel method of excitation for MEMS and optomechanical systems, this technique can be used in a broad range of future applications such as light-induced mechanical switching, line-of-sight wireless communication and solar energy harvesting.
